# Supplementary material for: Elucidating the role of human skeletal muscles in the pathogenesis of enterovirus D68 infection
Source: Life Sci Alliance. 2025 Sep 5;8(11):e202503372. doi: 10.26508/lsa.202503372 (PMC12413549; doi:10.26508/lsa.202503372)
Supplement: Supplementary file 1 [file LSA-2025-03372_TableS1.docx]

**Supplemental Table**

**Table S1**. List of primer sequences and concentrations used in this study. For degenerate nucleotide codes: Y = C or T; W = A or T; R = A or G; M = A or C; S = C or G; K = G or T; D = A, G or T; N = any base.

| **Amplicon** | **Primer** | **Primer pool** | **Nucleotide sequence (5' → 3')** | **Concentration of primer per reaction (µM)** |
| --- | --- | --- | --- | --- |
| 1 | Fwd 1A | Odd | AAAACAGCYTTGGGGTTGTTCC | 10 |
|  | Fwd 1B |  | TTAAAACAGCYTTGGGGTT | 10 |
|  | Rev 1 |  | TGCACATGRATYAAGAAACCAGA | 10 |
| 2 | Fwd 2A | Even | AGGTCAGTYAAATGGGAGGCTA | 25 |
|  | Fwd 2B |  | AAATCAGTYAAATGGGAAACTG | 25 |
|  | Rev 2A |  | GARGAYTCRYTGGGGACTATYA | 25 |
|  | Rev 2B |  | GAACCCTATCARRGAACATGT | 25 |
| 3 | Fwd 3A | Odd | TAGGYACRCATRTTGTTTGGGATTTT | 10 |
|  | Fwd 3B |  | ACYCCTCCAGGYGGRTCATG | 10 |
|  | Rev 3 |  | GCTAAGTGRTARTTRATTATTTT | 10 |
| 4 | Fwd 4 | Even | CACCAAATGCRCTCARTGCYAT | 10 |
|  | Rev 4 |  | CGGTTCAATGCGRGATTTGGAC | 10 |
| 5 | Fwd 5 | Odd | CAACAACAGAACAACARCAGGC | 10 |
|  | Rev 5A |  | CACCCTTTTCRGTYCTBGCAAT | 10 |
|  | Rev 5B |  | CTAAARCRTGTTCGRTATT | 10 |
| 6 | Fwd 6 | Even | AAACTTTTTGCTGGYATTCA | 25 |
|  | Rev 6 |  | ACTGARTCATTCAAGGARCTGG | 25 |
| 7 | Fwd 7A | Odd | TGGAYGARTAYATGGAAGA | 25 |
|  | Fwd 7B |  | TAGAACCRYTAGAYATYAG | 25 |
|  | Rev 7A |  | CCCCAAGTRRCCAAAATTTA | 25 |
|  | Rev 7B |  | ATCTRTTGAGATTRAYTYTGGG | 25 |
|  | Rev 7C |  | TCTARAACGAATCTAACCAT | 25 |
