# Supplementary material for: Elucidating the role of human skeletal muscles in the pathogenesis of enterovirus D68 infection
Source: Life Sci Alliance. 2025 Sep 5;8(11):e202503372. doi: 10.26508/lsa.202503372 (PMC12413549; doi:10.26508/lsa.202503372)
Supplement: Supplementary file 2 [file LSA-2025-03372_TableS2.docx]

**Supplemental Table**

**Table S2. List of RT-qPCR primer and probe sequences used in this study.**

| **Primer or probe** | **Nucleotide sequence (5' → 3')** |
| --- | --- |
| Forward primer | TGTTCCCACGGTTGAAAACAA |
| Reverse primer | TGTCTAGCGTCTCATGGTTTTCAC |
| Probe 1 | TCCGCTATAGTACTTCG |
| Probe 2 | ACCGCTATAGTACTTCG |
